# Short-Chain Fatty Acids Impair Neutrophil Antiviral Function in an Age-Dependent Manner

**DOI:** 10.3390/cells11162515

**Published:** 2022-08-13

**Authors:** Francisco J. Carrillo-Salinas, Siddharth Parthasarathy, Laura Moreno de Lara, Anna Borchers, Christina Ochsenbauer, Alexander Panda, Marta Rodriguez-Garcia

**Affiliations:** 1 Department of Immunology, Tufts University School of Medicine, Boston, MA 02111, USA; 2 Immunology Program, Tufts Graduate School of Biomedical Sciences, Boston, MA 02111, USA; 3 Immunology Unit, Biomedical Research Centre (CIBM), University of Granada, 18071 Granada, Spain; 4 Department of Medicine, Hem/Onc & CFAR, University of Alabama at Birmingham, Birmingham, AL 35233, USA; 5 Tufts Medical Center/Division of Pulmonary and Critical Care (PCCM), Boston, MA 02111, USA; 6 Tufts Clinical and Translational Science Institute (CTSI), Boston, MA 02111, USA

**Keywords:** short-chain fatty acids, neutrophil, HIV, aging, women

## Abstract

Half of the people living with HIV are women. Younger women remain disproportionally affected in endemic areas, but infection rates in older women are rising worldwide. The vaginal microbiome influences genital inflammation and HIV infection risk. Multiple factors, including age, induce vaginal microbial alterations, characterized by high microbial diversity that generate high concentrations of short-chain fatty acids (SCFAs), known to modulate neutrophil function. However, how SCFAs may modulate innate anti-HIV protection by neutrophils is unknown. To investigate SCFA-mediated alterations of neutrophil function, blood neutrophils from younger and older women were treated with SCFAs (acetate, butyrate and propionate) at concentrations within the range reported during bacterial vaginosis, and phenotype, migration and anti-HIV responses were evaluated. SCFA induced phenotypical changes preferentially in neutrophils from older women. Butyrate decreased CD66b and increased CD16 and CD62L expression, indicating low activation and prolonged survival, while propionate increased CD54 and CXCR4 expression, indicating a mature aged phenotype. Furthermore, acetate and butyrate significantly inhibited neutrophil migration in vitro and specifically reduced α-defensin release in older women, molecules with anti-HIV activity. Following HIV stimulation, SCFA treatment delayed NET release and dampened chemokine secretion compared to untreated neutrophils in younger and older women. Our results demonstrate that SCFAs can impair neutrophil-mediated anti-HIV responses.

## 1. Introduction

New human immunodeficiency virus (HIV) infections have been reduced by 40% since 1998, but around 1.5 million people were still newly infected with HIV in 2020 [1]. Although younger women are at higher risk in endemic areas, new HIV infections in older women are rising worldwide [2,3,4,5], a fact to take into account given the increase in the size of the elderly population expected in the upcoming decades [6,7]. 

The vaginal microbiota is a dynamic community of bacteria that works as a first-line defense against invading pathogens, along with the epithelial mucosal barrier and the immune mucosal response [8,9,10]. The vaginal microbiota, dominated by the *Lactobacilli* species that maintain high concentrations of lactic acid [11] and a low pH in the lower tract, is considered to be beneficial and reduce the risk of HIV acquisition [12,13,14,15]. However, multiple stimuli, including antibiotics, sexual activity, vaginal hygiene, menstrual cycle, and oral contraceptives, can alter these bacterial populations, resulting in vaginal dysbiosis and increased risk of HIV acquisition [12,16,17,18,19,20,21]. Data also indicate that the vaginal microbiome changes after menopause, with a decreased presence of *Lactobacillus* species compared to premenopausal women [22,23,24]. This fact, together with changes in immune cell responses, the pro-inflammatory environment, and a reduction of protective mediators in the female reproductive tract (FRT), may increase the risk of HIV acquisition in postmenopausal women [7,23,25,26,27,28].

An important consequence associated with vaginal dysbiosis is the increased presence of short-chain fatty acids (SCFAs), metabolic products of anaerobic bacterial fermentation. SCFAs are physiologically present in the genital mucosa at low concentrations (0–4 mM), with acetic, butyric and propionic acids being the most abundant [11,29,30,31,32]. However, under conditions that induce vaginal dysbiosis, the reduction in Lactobacilli is associated with a drop in lactate concentration and an increased vaginal pH that facilitates the growth of facultative and anaerobic bacteria, resulting in abnormally elevated concentrations of acetate, butyrate and propionate (20–140 mM) [11,29,30,31,32].

SCFAs present at mucosal surfaces act on epithelial cells to modify barrier function [9] and also diffuse into the subepithelial compartment to act directly on immune cells by interaction with the G-protein coupled receptors GPR43, GPR41 and GPR109A [33,34,35]. SCFAs have been described to play a critical role as immunomodulators to prevent mucosal inflammation in the gut [36]. However, high concentrations of SCFAs in the FRT seem to promote inflammation [9,31,37]. Genital inflammation is known to increase susceptibility to HIV infection [15,38,39], but very little is known about the effect of high concentrations of SCFAs on innate immune responses and the susceptibility to HIV infection in women [31].

We recently demonstrated that neutrophils from the FRT of healthy women release neutrophil extracellular traps (NETs) in response to HIV stimulation [40]. NET release is a process characterized by the extracellular ejection of DNA associated with granular proteins with antimicrobial activity [40,41,42], which has been shown to inactivate HIV in vitro [40,41]. In contrast, studies of inflammation in the context of sexually transmitted infections (STIs) describe associations between neutrophil-derived molecules in cervicovaginal secretions with an increased risk of HIV acquisition [43,44,45]. These apparently opposite findings highlight the gap in our knowledge about how alterations in the microbiome may affect neutrophil function and anti-HIV responses. Importantly, neutrophils highly express GPR43, the main SCFA receptor [35], representing a likely candidate to be modulated by changes in the microbial metabolome. However, it is unknown whether the anti-HIV potential of neutrophils is modified by SCFAs.

In this context, we hypothesize that pathological concentrations of SCFAs due to changes in genital microbiota can modulate neutrophil responses to HIV and directly impact the risk of HIV acquisition. To begin to answer this question, we optimized an in vitro model to evaluate the effects of pathological concentrations of SCFA on neutrophil function and anti-HIV activity in younger and older women.

We found that pathological concentrations of SCFAs reduced neutrophil activation in an age-dependent manner, inhibited neutrophil migration and reduced the release of NETs and innate antiviral molecules. Our findings provide proof of concept that genital microbial alterations that induce an increase in SCFA concentrations may impair neutrophils’ physiological functions and reduce their antiviral potential.

## 2. Materials and Methods

### 2.1. Study Subjects 

All investigations involving human subjects were conducted according to the principles expressed in the Declaration of Helsinki and carried out with the approval of the Institutional Review Board of Tufts University (protocol code: MODCR-01-11201, approved on 20 October 2014). Volunteer healthy and HIV-seronegative women were included in the study, and informed consent was obtained from all subjects. Information regarding age was provided, but no other information was disclosed. Women included in the study were classified as younger (*n* = 17; 18–28 years-old; median = 24) or older (*n* = 18; 65–72 years-old; median = 68).

### 2.2. Neutrophil Isolation from Human Peripheral Blood and Treatment with SCFAs

**Figure 1:**
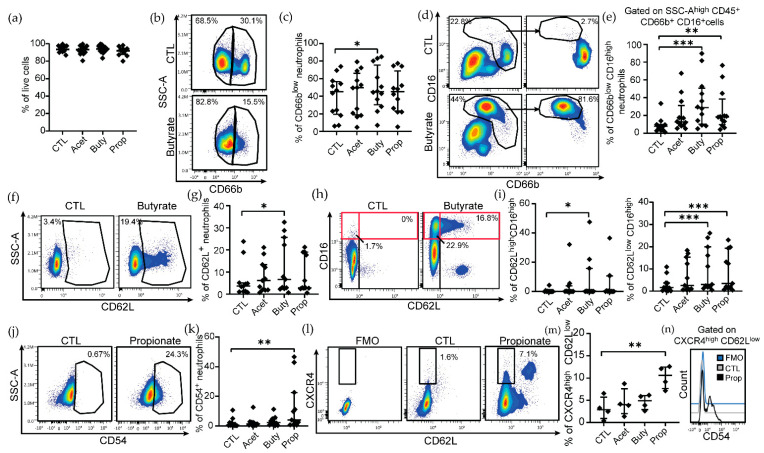
High concentrations of butyrate and propionate induce phenotypical changes in human neutrophils. (**a**) Quantification of cell death induced by high concentration of SCFA (*n* = 12). (**b**) Representative example of flow cytometry plots showing CD66b expression on neutrophils in control condition (CTL) and after butyrate treatment (25 mM) for 3 h. (**c**) Percentage of CD66b^low^ neutrophils after treatment with SCFAs (acetate, butyrate, and propionate) 25 mM for 3 h (*n* = 12). (**d**) Representative example of flow cytometry plots and (**e**) percentage of CD66b^low^ CD16^high^ neutrophils after SCFA treatment (*n* = 12). (**f**) Flow cytometry plots and (**g**) changes in percentage of CD62L^+^ neutrophils induced by SCFAs. Effect of pathological concentration of SCFAs on ((**h**,**i**); *n* = 11) CD16^high^ CD62L^high^ or CD62L^low^ neutrophil population and (**j**,**k**) CD54^+^ neutrophils (*n* = 11). (**l**) Representative flow cytometry plots and (**m**) quantification of the percentage of CXCR4^high^ CD62L^low^ neutrophils after treatment with pathological concentration of SCFAs (*n* = 4). (**n**) Changes induced by propionate treatment in CD54 expression of neutrophils gated on CXCR4^high^ CD62L^low^ population. Each dot represents a different patient (age of patients: 18–72 years old). Non-parametric paired Friedmann test was used, * *p* ≤ 0.05, ** *p* ≤ 0.01, *** *p* ≤ 0.001. CTL: control; Acet: acetate 25 mM; Buty: butyrate 25 mM; Prop: propionate 25 mM.

Venous blood of healthy women was collected into 10 mL EDTA tubes (BD Vacutainer; Franklin Lakes, NJ, USA). Polymorphonuclear cell (PMN) isolation was performed by positive selection using CD15 MicroBeads (Miltenyi Biotec; Auburn, CA, USA) and a whole blood column kit following the manufacturer’s instructions (Miltenyi Biotec). This isolation method was effective with 92.84% viability (Figure 1a and Figure S1a) and >94% neutrophil enrichment (as CD45^+^ CD15^+^ CD66b^+^ cells), determined by flow cytometry. Purified neutrophils were resuspended in HBSS culture medium (Hanks’ Balanced Salt Solution, Gibco; Waltham, MA, USA) for imaging analysis or in X-VIVO 15 media for cell culture (Lonza; Bend, OR, USA) and stimulated with 25 mM of sodium acetate, sodium butyrate or sodium propionate for 1 or 3 h as indicated for further analysis.

### 2.3. Determination of Neutrophil Phenotype by Flow Cytometry

Neutrophils were fixed with 4% PFA for 30 min at 4 °C, washed and stained for 20 min in the dark with the following anti-human antibodies: CD45-APC-Cy7 (clone 2D1; Biolegend, San Diego, CA, USA), CD54-BV421 (clone HA58; Biolegend), CD62L-BV711 (clone SK11; Biolegend), CXCR4-BV-785 (clone 12G5; Biolegend), CD66b-APC (clone REA306; Miltenyi Biotec), CD15-FITC (Miltenyi Biotec) and CD16-PE (clone 3G8; BD Biosciences, Franklin Lakes, NJ, USA). A live/dead fixable blue dead cell stain kit (Thermo Scientific; Waltham, MA, USA) was used to assess cell death in cultures before fixation. Fluorescence Minus One (FMO) controls were used to identify and gate positive populations (Figure 1 and Figure S1b). Analysis was performed on an LSRII flow cytometer (BD; Ashland, Wilmington, DE, USA) or Aurora cytometer (Cytek Biosciences; Fremont, CA, USA) and assessed using FlowJo software (BD) or OMIQ (www.omiq.ai (accessed on 13 July 2021)). The expression of surface markers was measured by the percentage of positive cells.

### 2.4. Migration Assay

Neutrophil migration was evaluated using a Transwell assay inserted into an ultra-low attachment 24-well plate (Corning Inc., Corning, NY, USA). Cells were seeded at a density of 4 × 10^5^/well in X-VIVO 15 media (Lonza) into the upper chamber of a Transwell insert (5 µm pore size; Corning, Inc.), and XVIVO-15 with sodium acetate, sodium butyrate or sodium propionate at a final concentration of 25 mM was added to the lower chamber to study if SCFAs at this concentration could act as a chemoattractant. After 3 h at 37 °C, the cells from both chambers were collected and stained for immune phenotyping by flow cytometry. The migration ratio was calculated by dividing the number of cells in the bottom chamber by the sum of cells in the top + bottom chamber and normalizing to the control group.

### 2.5. Generation of GFP-Labeled VLPs

Modified pNL43 provirus-based plasmid for expression of GFP-labeled viral-like particles (VLPs) and encoding NL43 Env in cis (referred to as pNL4GagGSGFP/K795) was described previously [46]. Briefly, the enhanced GFP (EGFP) coding sequence is expressed in the frame at the 3’end of the gag, replacing the protease and most of the reverse transcriptase coding region. The Ψ-signal on the RNA and the complete gag open reading frame (ORF) remain intact. Furthermore, a plasmid with an inactivated Env ORF, resulting in no expression of functional Env protein (referred to as pNL4GagGSGFPDelta-env/K806), was derived from K795 for pseudotyping and complemented with pBaL.26 Env expression plasmid (NIH AIDS Reagent program, catalog number 11,446, contributed by Dr. John Mascola) [47]. Non-infectious, EGFP-labelled VLPs were produced by transfection, concentrated by ultracentrifugation, and enumerated essentially as described [46].

### 2.6. Time-Lapse Microscopy of NETs

Human purified neutrophils from blood were plated in a 96-well plate (Corning Inc.; Corning, NY, USA) and stimulated with 25 mM of sodium acetate, sodium butyrate or sodium propionate (Sigma-Aldrich; St. Louis, MO, USA), in the presence or absence of GFP-labeled HIV-viral like particles (HIV-VLPs). Cytotox red reagent (Essen Bioscience; Ann Arbor, MI, USA) was used to label DNA. Images were collected every 3–5 min for at least 3 h at 37 °C using a 10x objective with the IncuCyte S3 (Sartorius; Bohemia, NY, USA). Extracellular DNA-labeled red signal and GFP-VLP signal were quantified to determine the NET-HIV area with the Incucyte software as described [40]. 

### 2.7. HIV Stimulation

HIV-1-BaL (R5) isolates were obtained from the AIDS Research and Reference Reagent Program, Division of AIDS, NIAID, NIH, from Dr. Suzanne Gartner, Dr. Mikulas Popovic and Dr. Robert Gallo [48] and propagated in PBMCs as described [49]. Purified blood neutrophils were stimulated with HIV-1 BaL for 1h at an MOI of 0.5, after which the culture supernatants were collected and stored at −80 °C until used for cytokine and chemokine analysis by Luminex. Uninfected controls were incubated for the same length of time in media without the virus.

### 2.8. Quantification of Cytokines and Chemokines by Luminex

Supernatants from SCFA- and HIV-stimulated neutrophils were centrifuged at 18,000× *g* for 10 min to remove any cell debris and NETs. Then, supernatants were transferred to a new plate for HIV inactivation with 0.5% Triton X-100 (Sigma) for 30 min at 4 °C. Two different panels of cytokines and chemokines were measured using Millipore human cytokine multiplex kits (EMD Millipore Corporation; Billerica, MA, USA) following the manufacturer’s instructions. Panel 1: sCD40L, MIP-1α, MIP-1β, GM-CSF, IFNγ, TNFα, IL-1β, IL-5, IL-6, IL-8, IL-10, IL-22, GROα, IFNα2, IL-13, IL-27 and PDFG-AB/BB. Panel 2: IL-8, MCP-1, MIP-1α, RANTES, MDC and MIG. Signal was measured using the MAGPIX system by Luminex (Luminex Corporation; Austin, TX, USA) and quantified with Luminex xPONENT software.

### 2.9. ELISA

The concentration of α-defensins 1–3 was quantified in cell-free culture supernatants after 3 h of treatment with acetate, butyrate or propionate 25 mM using the commercial Human alpha-defensin 1 DuoSet ELISA (R&D Systems; Minneapolis, MN, USA) following the manufacturer’s instructions. The control group was incubated with X-VIVO 15 media. The concentration of sCD62L was also measured using the Human L-Selectin/CD62L DuoSet ELISA (R&D Systems) from the same supernatants used for α-defensin and Luminex.

### 2.10. Quantification of GPR43 by Western Blot

Human neutrophil pellets were resuspended in extraction buffer containing RIPA buffer, 1% NP-40, 1mM PMSF, 1× phosphatase inhibitor (PhosSTOP, Millipore Sigma; Burlington, MA, USA), and 1× protease inhibitor (cOmplete, Millipore Sigma) and were lysed for 45 min on ice. The cell lysate was centrifuged at 12,000× *g* at 4 °C for 5 min. Protein concentration was determined with a protein assay reagent (Pierce 660 nm, Thermo Scientific), and 10–20 µg were mixed with 1× Laemmli SDS sample buffer and heated at 95 °C for 5 min. Samples were run in 4–20% acrylamide gels (Mini-PROTEAN TGX Precast Protein Gels, Bio-Rad; Hercules, CA, USA) at 200 V for 1 h. After electrophoresis, proteins were transferred to a PVDF membrane using a rapid transfer system (Trans-Blot Turbo Transfer System, Bio-Rad). Membranes were blocked using a blocking solution (Pierce Fast Blocking Buffer, Thermo Scientific) for 5 min at RT, washed with 0.1% Tween20 in TBS (pH 7.6), and then incubated overnight with 1:1000 polyclonal rabbit anti-human GPR43 antibody (Thermo Scientific) in a 5% BSA, 0.05% NaN3, 0.1% Tween-20 TBS solution. After washing, the membrane was incubated with 1:7500 anti-Rabbit IgG (H + L) (IRDye 800CW, Li-Cor; Lincoln, Dearborn, MI, USA) in a 1% dry milk, 0.1% Tween 20, TBS solution for 45 min at RT. Protein quantification was performed using the Li-Cor Odyssey system. Protein levels were relativized to unstimulated control.

### 2.11. Statistical Analysis

Data analysis was performed using the GraphPad Prism 9 software. Data are represented as median ± interquartile range (IQR). A two-sided *p*-value ≤ 0.05 was considered statistically significant. Non-parametric Mann–Whitney U test or Wilcoxon’s matched pair test was used for comparison of two groups, and non-parametric Kruskal-Wallis or Friedman tests followed by Dunn’s post-test were used for comparison of three or more groups. * *p* ≤ 0.05; ** *p* ≤ 0.01; *** *p* ≤ 0.001. Grubb’s analysis (alpha = 0.05) was used to identify potential outliers.

## 3. Results

### 3.1. Pathological Concentrations of SCFAs Induce Phenotypical Changes in Human Blood Neutrophils

In order to identify phenotypical changes in neutrophils under conditions of high concentration of SCFAs, we incubated blood neutrophils from healthy women (range of age: 18–72 years old) with three different SCFAs (butyrate, propionate or acetate) at a pathological concentration (25 mM) and compared the expression of several activation markers by flow cytometry (Figure S1a; gating strategy). This concentration was selected based on previous literature indicating a range concentration of SCFA of 20–140 mM during vaginal dysbiosis [11,29,30,31,32]. First, we observed that pathological concentrations of SCFAs did not induce cell death in neutrophils after 3 h of treatment compared to the control group (Figure 1a). In untreated neutrophils, two subpopulations were identified based on CD66b expression, CD66b^low^ and CD66b^high^ (Figure 1b, top panel). Treatment with butyrate specifically induced a significant decrease in the expression of CD66b (Figure 1b; bottom panel), increasing the proportion of CD66b^low^ neutrophils (Figure 1c), while no significant changes were observed for CD66b expression when neutrophils were treated with acetate or propionate (Figure 1c). In addition, butyrate and propionate treatment modified CD16 expression, inducing a shift from the CD66b^high^CD16^low^ population found in untreated neutrophils (Figure 1d; top panels) to CD66b^low^CD16^high^ in butyrate and propionate-treated neutrophils (Figure 1d,e; bottom panels). Butyrate treatment also increased CD62L expression on neutrophils (Figure 1f,g), while no significant changes were detected for the other SCFAs (Figure 1g). 

Because CD16 and CD62L can define different neutrophil subsets with distinct effector functions [50], we next determined changes in the co-expression of these two markers (Figure 1h). Consistent with our observations with each individual marker, we detected a significant increase in the CD62L^high^CD16^high^ neutrophil population after butyrate treatment (Figure 1h,i; left graph), indicating a mature and partially activated phenotype. Although we identified an outlier in Figure 1i (left graph), the difference remained significant after excluding from the analysis this outlier data point in the data set (*p* = 0.016). In addition, butyrate and propionate treatment also increased the proportion of CD62L^low^ CD16^high^ neutrophils (Figure 1i; right graph). 

Finally, we analyzed the expression of CD54 (intracellular adhesion molecule 1, ICAM-1) in neutrophils, a marker of activation and migration. Only propionate induced a significant increase in the proportion of CD54^+^ neutrophils (Figure 1j,k), while no effect was observed with the other SCFAs. Since upregulation of CD54 and downregulation of CD62L in combination with CXCR4 [51] are markers that indicate “aged” neutrophils, an overly active population of circulating neutrophils with an expanded lifespan, we further explored the expression of CXCR4 following SCFA treatment to confirm the induction of an aged phenotype. Only pathological concentrations of propionate significantly increased the proportion of CXCR4^high^CD62L^low^ neutrophils (Figure 1l,m), which also showed higher expression of CD54 (Figure 1n), characteristic of neutrophils with an aged phenotype [51].

Taken together, our results suggest that butyrate at pathological concentrations reduces activation of neutrophils and increases maturation, while propionate induces phenotypical alterations characteristic of “aged” neutrophils.

### 3.2. Effects of SCFA Treatment Are Enhanced in Neutrophils from Older Women

**Figure 2:**
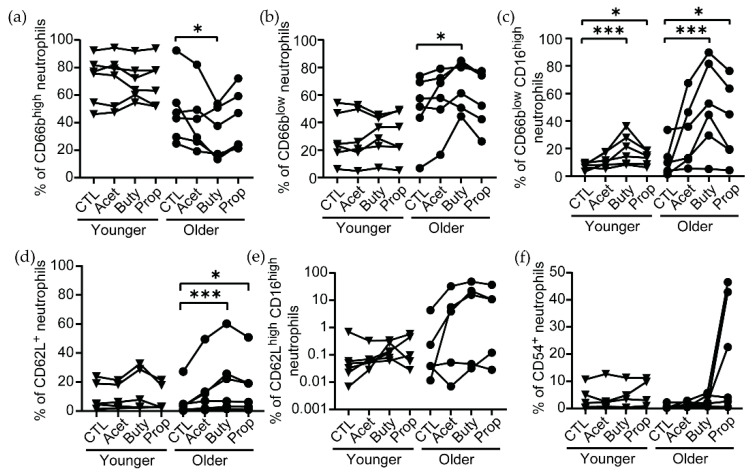
Pathological concentrations of butyrate and propionate preferentially modifies the phenotype of neutrophils from older women. Effect of SCFAs on the percentage of (**a**) CD66b^high^, (**b**) CD66b^low^, (**c**) CD66b^low^CD16^high^, (**d**) CD62L^+^, (**e**) CD62L^high^CD16^high^, and (**f**) CD54^+^ neutrophils after 3 h of treatment (25 mM) in neutrophils from younger (triangles) and older women (circles), quantified by flow cytometry. Each dot represents a different patient (younger = 6; older = 5–6). Non-parametric paired Friedmann test was used, followed by Dunn’s post-test for multiple comparisons; * *p* ≤ 0.05, *** *p* ≤ 0.001. CTL: control; Acet: acetate 25 mM; Buty: butyrate 25 mM; Prop: propionate 25 mM.

Recognizing that as women age, immune functions and the composition of the vaginal microbiome are modified [7,24], we stratified the women in our study into younger (average of 24.78 years old) and older groups (average of 66.22 years old) and evaluated phenotypical changes to determine if age had any potential effects on susceptibility to a pathological concentration of SCFAs. Interestingly, the CD66^high^ (Figure 2a) and CD66b^low^ (Figure 2b) neutrophil populations were very conserved in younger women and did not change after treatment with SCFAs. In contrast, older women showed high variability in the levels of CD66b expression, and these levels were significantly reduced after butyrate treatment, with a decrease in the proportion of CD66b^high^ neutrophils (Figure 2a) and an increase in CD66b^low^ neutrophils (Figure 2b). Furthermore, the observed effect of butyrate and propionate increasing CD16 expression on CD66b^low^ neutrophils (Figure 1c,d) was enhanced in neutrophils from older women (butyrate: 12.2-fold change, propionate: 8-fold change) compared to younger women (butyrate: 3-fold change, propionate: 2-fold change) (Figure 2c). Similarly, butyrate and propionate treatment only increased CD62L expression on neutrophils in older women, with no significant changes in younger women (Figure 2d). However, when we analyzed changes in the co-expression of CD62L and CD16, we observed high variability of a CD62L^high^ CD16^high^ neutrophil population in older women after SCFA treatment, while this population was almost absent in younger women (Figure 2e). 

Finally, although propionate induced an increase in the proportion of CD54^+^ neutrophils in the older population, this change did not reach statistical significance (Figure 2f).

Collectively, our data indicate that neutrophils from older women are more responsive to high concentrations of SCFAs than younger women, specifically to butyrate and propionate, resulting in a non-activated mature phenotype. 

### 3.3. Pathological Concentrations of SCFAs Inhibit Neutrophil Migration In Vitro

**Figure 3:**
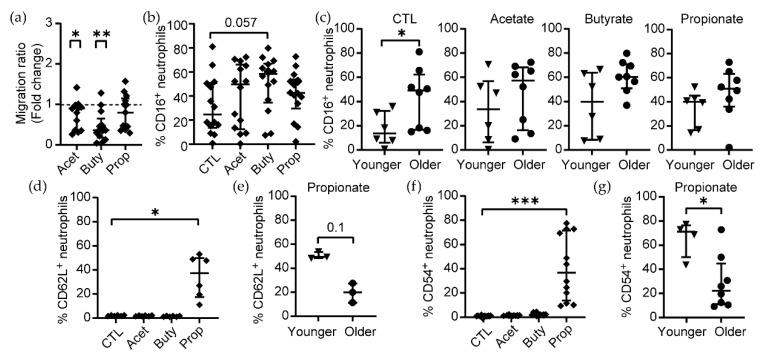
High concentrations of acetate and butyrate inhibit neutrophil migration. (**a**) Migration ratio of neutrophils in the presence of SCFAs 25 mM for 3 h (*n* = 14). (**b**) Expression of CD16 on neutrophils that migrated to the bottom chamber after 3 h in the absence (control: CTL) or presence of different SCFAs (25 mM) measured by flow cytometry (*n* = 14). (**c**) Effect of age on CD16^+^ neutrophils treated with SCFAs 25 mM (younger = 6; older = 8). Migrated neutrophils in the bottom chamber of the transwell were collected and the percentage of (**d**) CD62L^+^ neutrophils in the absence or presence of SCFAs was quantified (*n* = 6). (**e**) Effect of age on CD62L^+^ neutrophils that migrated towards propionate (younger = 3; older = 3). The same analysis was conducted for CD54^+^ neutrophils (**f**, *n* = 12), and effect of age on CD54^+^ neutrophils that were chemoattracted by propionate (**g**) was examined (younger = 4; older = 8). Each dot represents a different patient. (**a**,**c**) Wilcoxon t-test and (**b**,**d**–**g**) non-parametric paired Friedmann test were used; * *p* ≤ 0.05, ** *p* ≤ 0.01, *** *p* ≤ 0.001. CTL: control; Acet: acetate 25 mM; Buty: butyrate 25 mM; Prop: propionate 25 mM.

CD62L (L-selectin) and CD54 (ICAM-1) are adhesion molecules involved in neutrophil transmigration [52]. Since we observed changes in CD62L and CD54 expression following SCFA treatment, we next investigated neutrophil migration in the context of pathological concentrations of SCFAs. To determine if pathological concentrations of SCFAs would act as a chemoattractant for neutrophils, we used a transwell system, plating neutrophils on the top chamber and a high concentration of SCFAs (25 mM) or control media in the bottom chamber, and the phenotype of neutrophils was determined by flow cytometry following the same gating strategy used for Figure 1, Figure 2 and Figure S1a. After 3 h, high concentrations of acetate and butyrate significantly inhibited neutrophil migration compared to the control condition, while propionate did not affect neutrophil migration compared to control media (Figure 3a). 

Next, we analyzed changes in CD16, CD62L and CD54 expression in the neutrophils that migrated. We observed a trend towards an increased proportion of CD16^+^ neutrophils in the butyrate condition, although it did not reach statistical significance compared to untreated controls (Figure 3b). When we evaluated the effects of age, untreated neutrophils from older women showed significantly higher expression of CD16 after migration compared to neutrophils from younger women (Figure 3c). However, this difference was abrogated in the presence of SCFAs (Figure 3c).

In contrast, we found a significant increase in CD62L^+^ neutrophils after migration in the propionate condition (Figure 3d), which was independent of age (Figure 3e), while these populations were absent in the acetate and butyrate conditions. In addition, we observed a higher proportion of CD54^+^ neutrophils after migration to propionate (Figure 3f), and this CD54^+^ neutrophil population was significantly more abundant in younger compared to older women (Figure 3g).

Taken together, these results demonstrate that high concentrations of acetate and butyrate inhibit neutrophil migration, while propionate does not affect migration capacity but modifies the phenotype of migrated neutrophils with increased expression of CD62L and CD54 in an age-dependent manner.

### 3.4. High Concentrations of SCFAs Modify Innate Secretion Profile by Neutrophils from Older Women

**Figure 4:**
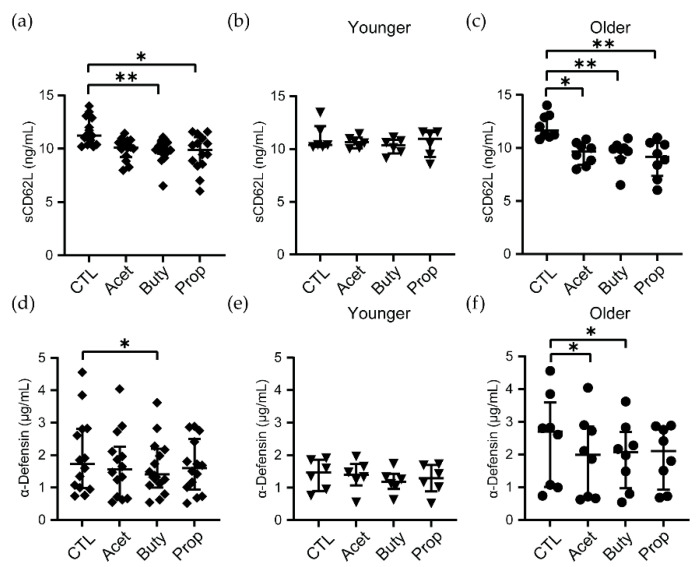
Pathological concentrations of SCFAs significantly reduced the release of sCD62L and α-defensin 1–3 by neutrophils from older women. (**a**) Quantification of sCD62L released by SCFA-treated neutrophils and stratified by younger (**b**) and older women (**c**), measured by ELISA. (**d**) Quantification of α-defensin 1–3 released by SCFA-treated neutrophils and stratified by younger (**e**) and older women (**f**) after 3 h, measured by ELISA. Each dot represents a different patient (*n* = 14; younger = 6, older = 8). Non-parametric paired Friedmann test was used; * *p* ≤ 0.05, ** *p* ≤ 0.01. CTL: control; Acet: acetate 25 mM; Buty: butyrate 25 mM; Prop: propionate 25 mM.

Following cellular activation and migration, CD62L is rapidly cleaved off and released, displaying immunomodulatory properties [53], mainly inhibiting leukocyte recruitment. Because we detected changes in activation phenotype and surface expression of CD62L after SCFA treatment of neutrophils, we next measured levels of soluble CD62L (sCD62L) in supernatants. The concentration of sCD62L was significantly decreased when neutrophils were treated with butyrate or propionate for 3 h, but not acetate (Figure 4a). Interestingly, when samples from women were stratified by age, SCFAs did not alter sCD62L secretion in neutrophils from younger women (Figure 4b), but sCD62L was significantly reduced after acetate, butyrate and propionate treatment in older women compared to untreated controls (Figure 4c).

To evaluate if other innate secreted molecules could also be affected by SCFA treatment, we measured the secretion of α-defensin 1–3, an antimicrobial peptide very abundant in neutrophils that displays broad spectrum microbicidal activity, including anti-HIV activity [54]. When all women were analyzed together, only butyrate significantly reduced α-defensin 1–3 release by neutrophils after 3 h (Figure 4d). After separating women based on age, no changes were observed in the younger group (Figure 4e), while acetate and butyrate treatments significantly reduced α-defensin 1–3 release by neutrophils from older women (Figure 4f). 

Next, we determined if classical pro-inflammatory molecules were modified by SCFA treatment. A panel of cytokines and chemokines was measured in supernatants from SCFAs-treated neutrophils by Luminex. However, none of the secreted cytokines and chemokines analyzed, which included the classical pro-inflammatory cytokines GM-CSF, IFN-γ, IL-1β, IL-6, IL-8 and TNFα, changed after 3 h of treatment with SCFAs (Figure S2). No significant differences were found between younger and older women, although neutrophils from older women showed a trend to secrete lower levels of most of the molecules analyzed (Figure S2). 

Overall, these data demonstrate that pathological concentrations of SCFAs modify secretion profiles of innate molecules selectively in neutrophils from older women but do not alter the secretion of pro-inflammatory cytokines and chemokines.

### 3.5. High Concentrations of SCFAs Delay NET Release and Chemokine Secretion in Response to HIV

**Figure 5:**
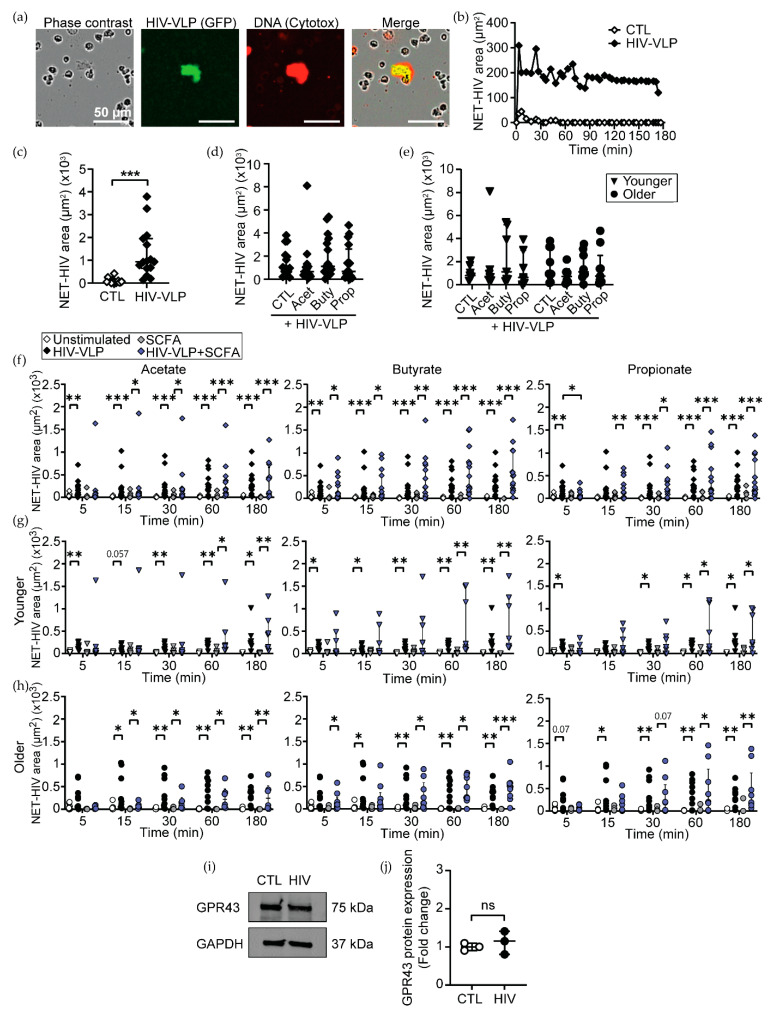
Pathological concentrations of SCFA induce a significant delay in HIV-induced NET release by neutrophils. (**a**) Representative image of NET formation and co-localization with HIV-VLPs, quantified with IncuCyte S3 system. (**b**) Total NET-HIV area of unstimulated controls (white dots) and HIV-VLP (black dots) is represented over time. (**c**) Quantification of HIV-NET area in the first 3 h and (**d**) after treatment with 25 mM of acetate, butyrate or propionate after stimulation with HIV-VLPs (*n* = 16). (**e**) Effect of age on NET release by SCFA-treated neutrophils. (**f**) Comparison of HIV-induced NET release by time intervals in all women (*n* = 16) and separated in (**g**) younger (*n* = 7) and (**h**) older (*n* = 9) groups. (**i**) Representative Western blot and (**j**) neutrophil GPR43 protein quantification after 1 h of stimulation with HIV-VLPs, relative to unstimulated CTL (*n* = 3). Scale bar: 50 µm. Each dot represents a different patient (*n* = 16; younger = 7; older = 9). Wilcoxon’s matched-pairs signed-rank test was used for two-group comparisons, and Kruskall–Wallis with Dunn’s post-test was used to compare three or more groups. * *p* ≤ 0.05, ** *p* ≤ 0.01, *** *p* ≤ 0.001. CTL: control; Acet: acetate 25 mM; Buty: butyrate 25 mM; Prop: propionate 25 mM.

Since vaginal dysbiosis is known to increase the vaginal concentrations of SCFAs [55,56] and the risk of HIV acquisition [13,17,19,21], we next investigated if the high SCFA environment could affect neutrophil antiviral response to HIV. SCFA-treated neutrophils from younger and older women were challenged with HIV-VLPs, and the release of NETs was measured with time-lapse microscopy as described [40]. Consistent with our previous results, we observed that following HIV stimulation, neutrophils actively released NETs, which entrapped HIV-VLPs (Figure 5a). NET release started within minutes after HIV exposure and was sustained for at least 3 h after HIV stimulation (Figure 5b). Quantification of NET-HIV complexes demonstrated a significant upregulation after HIV exposure compared to unstimulated controls (Figure 5c).

Next, we asked if SCFA could modify NET release by themselves or in response to HIV. In the absence of HIV stimulation, high concentrations of acetate, butyrate or propionate did not increase NET release in comparison to control untreated neutrophils (Figure S3). In addition, pathological concentrations of SCFAs did not modify the overall magnitude of HIV-induced NET release during the first 3 h after stimulation, whether women were analyzed together (Figure 5d) or separated into younger and older groups (Figure 5e). 

Next, we evaluated if SCFA treatment could modify the timing of NET release. HIV-induced NETs were significantly upregulated as early as 5 min following HIV stimulation in untreated and butyrate-treated neutrophils (Figure 5f, white and black symbols). In contrast, acetate- and propionate-treated neutrophils showed a 15 min delay in NET release after HIV stimulation (Figure 5f). Furthermore, propionate-treated neutrophils not only delayed but significantly decreased initial NET release following HIV stimulation compared to the HIV control condition (Figure 5f). Then, we evaluated whether SCFA treatment affected neutrophils from younger and older women differently. In the absence of SCFAs, HIV-induced NET release was detected 5 min after stimulation in neutrophils from younger women (Figure 5g) and 15 min after stimulation in neutrophils from older women (Figure 5h). Interestingly, acetate, butyrate and propionate treatments delayed 1 h HIV-induced NET release in neutrophils from younger women (Figure 5g), while butyrate induced a 30 min delay and propionate induced a 1h delay in the anti-HIV response of old neutrophils (Figure 5h).

As a control, we determined whether HIV could modify the protein level of the main receptor for SCFAs, GPR43, which is highly expressed by neutrophils [35]. HIV did not change the expression of GPR43 in neutrophils after 1h of stimulation, quantified by Western blot (Figure 5i,j).

**Figure 6:**
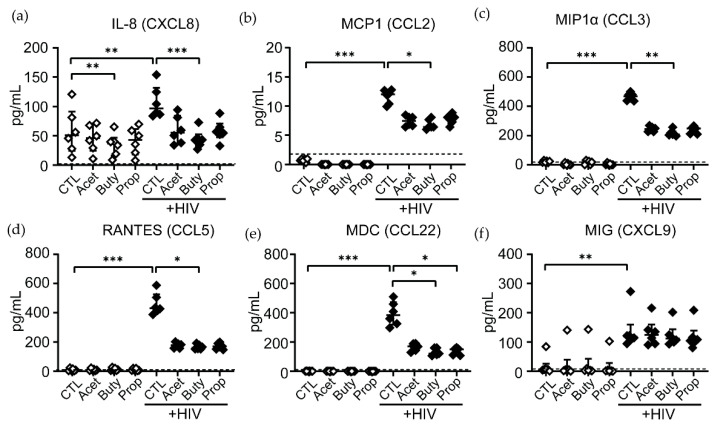
High concentrations of butyrate significantly reduces the release of chemokines in response to HIV stimulation. Neutrophils were stimulated with HIV-BaL in the presence or absence of SFCAs for 1 h, and cell-free supernatants were used to quantify the concentration of the following molecules by Luminex: (**a**) IL-8 (CXCL8), (**b**) MCP-1 (CCL2), (**c**) MIP1α (CCL3), (**d**) RANTES (CCL5), (**e**) MDC (CCL22) and (**f**) MIG (CXCL9). Each dot represents a different patient (*n* = 6). Dotted line: limit of detection. Non-parametric paired Friedmann test was used, * *p* ≤ 0.05, ** *p* ≤ 0.01, *** *p* ≤ 0.001. CTL: control; Acet: acetate 25 mM; Buty: butyrate 25 mM; Prop: propionate 25 mM.

In order to study if additional mechanisms involved in neutrophil-mediated anti-HIV responses were altered by a high concentration of SCFAs, neutrophils were stimulated with replication-competent HIV-BaL for 1 h and a selected panel of chemokines relevant for chemoattraction of HIV-target cells, and direct anti-HIV activity was measured in supernatants by Luminex [39]. We observed a significant increase in the release of IL-8 (CXCL8), MCP-1 (CCL2), MIP1α (CCL3), RANTES (CCL5), MDC (CCL22) and MIG (CXCL9) by neutrophils in response to HIV (Figure 6), while other cytokines, such as TNFα or IL-10, were undetectable (data not shown). Interestingly, HIV stimulation in the presence of a high concentration of SCFAs dampened the release of these molecules, with a significant reduction for most molecules detected in the presence of butyrate and a significant reduction in MDC secretion in the presence of butyrate and propionate (Figure 6). 

Taken together, our findings indicate that a pathological concentration of SCFAs significantly delays HIV-induced NET release by neutrophils from both younger and older women and reduces the release of chemokines in response to HIV stimulation. 

## 4. Discussion

Our study demonstrates that pathological concentrations of SCFAs modify neutrophil activation, secretion profile and anti-HIV responses in an age-dependent manner. We found that different SCFAs exert specific effects and that neutrophils from older women are more susceptible to modulation by SCFA treatment. Our findings are relevant to understanding how changes in the composition of the genital microbiome that alter the metabolome may affect neutrophil-mediated innate protection in the genital tract of younger and older women. 

It is well known that women with vaginal dysbiosis and bacterial vaginosis (BV) have a shift in their vaginal microbiota, with an increased number and diversity of facultative and anaerobic bacteria [8]. Furthermore, high-diversity bacterial communities in the FRT are strongly associated with pro-inflammatory genital cytokines that activate immune cells in vivo [15,57]. Several studies have shown that there is a shift in metabolites from lactate toward mixed SCFAs during vaginal dysbiosis [30,31,55,56,58], which include a wide range in concentrations (20–140 mM) of acetate, butyrate and propionate [11,29,30,31]. 

To understand how changes in the microbiome and microbial metabolites may affect neutrophil-mediated innate protection against infections, in this study, we optimized an in vitro model to evaluate the potential effects of a high-SCFA-concentration environment on neutrophils by treating blood neutrophils with three different SCFAs (acetate, butyrate and propionate), known to be increased in conditions with vaginal dysbiosis and at concentrations described as pathological in the lower tract of the FRT [9,11,29,30]. Under these conditions, we observed modifications specific to each SCFA. Butyrate induced phenotypical changes in neutrophils, with decreased CD66b and increased CD16 and CD62L expression. CD66b is an adhesion molecule and a marker of neutrophil activation [59], while CD16 is involved in neutrophil survival [60], and CD62L is a marker for transmigration and maturation that is downregulated as neutrophils age [61,62]. Therefore, our results suggest that a high concentration of butyrate induces low activation and longer survival of non-aged mature neutrophils. Consistently, previous studies have shown upregulation of CD16 expression on vaginal neutrophils in women with BV [63] and inhibition of neutrophil apoptosis after treatment of peripheral neutrophils with high concentrations of butyrate in vitro [64]. Although Aoyama et al. [65] reported a significantly higher percentage of neutrophil apoptosis after treatment with a high concentration of butyrate, this only happened after 20 h in culture, and no differences were observed only after 3 h, which is in line with our observations. Furthermore, propionate treatment very specifically upregulated CD54 (ICAM-1) expression, which is typically upregulated in transmigrating neutrophils but not in circulating or tissue-resident neutrophils [66]. 

Recently, aged neutrophils have been considered important in inflammatory responses [51]. This pro-inflammatory subset, defined as CXCR4^high^ CD62L^low^ CD54^high^ neutrophils, displays increased capacity to phagocytize and migrate [51], but can mediate tissue damage by producing NETs and reactive oxygen species (ROS) under conditions of sterile inflammation [67]. In this line, preventing the recruitment of aged neutrophils has been demonstrated to be protective against tissue damage [62]. Interestingly, we observed that pathological concentrations of propionate induced a phenotype of aged neutrophils. A previous unpublished study has reported a higher number of aged neutrophils in cervicovaginal fluid and cervical cytobrush in women with vaginal dysbiosis [68], which could be deleterious for the genital mucosa under vaginal dysbiosis.

Interestingly, phenotypical changes were mainly observed in older women in the presence of SCFAs, while neutrophils from younger women only showed a minimal increase in a CD66^low^ CD16^high^ population after butyrate and propionate treatment, suggesting enhanced sensitivity to the effects of SCFA with aging. The reason for this remains unsolved, but it could be related to changes in the expression of SCFA receptors. Future studies are needed to address this question. 

Upon neutrophil activation, surface expression of CD62L is quickly reduced mainly through proteolytic cleavage [53,69], which results in the release of a functionally active soluble extracellular fragment, known as sCD62L. sCD62L is detected in the plasma of healthy humans [70,71] and plays two major roles: preventing lymphocyte recirculation [72] and inhibiting transendothelial migration of other leukocytes [71]. Consistent with the increase in expression of membrane-bound CD62L in neutrophils from older women after SCFA treatment, we detected a significant reduction in the release of sCD62L. Given that sCD62L has been involved in the regulation of leukocyte adhesion and migration, the functional consequences of reduced sCD62L in the FRT of older women remain to be investigated.

In addition to changes in phenotype, we observed reduced migration of neutrophils towards SCFA-rich environments in both younger and older women. Particularly, high concentrations of acetate and butyrate, but not propionate, significantly inhibited neutrophil migration. These findings are in agreement with previous studies with human peripheral neutrophils [35] and animal models [73,74,75], demonstrating that pathological concentrations of SCFAs inhibit neutrophil migration. Interestingly, while propionate did not affect neutrophil migration, we describe for the first time that unstimulated neutrophils which migrated towards propionate experienced phenotypical changes with upregulation of CD54 in an age-dependent manner, suggesting that these neutrophils are prepared for transendothelial migration. 

Taken together, our study and previous studies from others suggest that physiological levels of SCFAs can act as chemoattractants for immune cells and specifically for neutrophils [35,76], while pathological concentrations prevent neutrophil migration independently of age. This anti-chemotactic action could be a contributing factor to the described low presence of neutrophils in vaginal secretions from women with BV [11,37,77], although reduced neutrophil presence during BV has not been confirmed in all studies [10,63]. Importantly, while our in vitro model evaluated the individual effects of each SCFA, neutrophils in the genital tract would be exposed to a combination of SCFAs, and therefore, the overall result remains to be elucidated. However, given that both butyrate and acetate inhibited migration, we speculate that SCFAs in combination at pathological concentrations would likely inhibit neutrophil recruitment as a final result. Nevertheless, it will be important to determine which combinations and specific concentrations of SCFAs attract or inhibit neutrophil chemotaxis in vivo since some microbial alterations associated with STIs are accompanied by an increased presence of neutrophils in genital secretions, while other alterations are not.

SCFAs are known to play an important role in the host as immunomodulators [36]. Despite their well-described anti-inflammatory properties at physiological levels, SCFAs are less inhibitory when found at high concentrations [9,31,74,78]. Previous studies have reported that peripheral blood mononuclear cells (PBMCs) increased their production of pro-inflammatory cytokines, including IL-1β, IL-6 and IL-8, after 6 to 18 h of treatment with pathological concentrations of SCFAs alone [31]. In contrast to these findings, we did not observe an increase in classical pro-inflammatory cytokines following SCFA treatment for 3 h, suggesting that pathological levels of SCFAs do not enhance the pro-inflammatory secreted profile of neutrophils. We did observe, however, that butyrate and acetate treatment reduced the secretion of α-defensin 1–3, particularly in neutrophils from older women. α-Defensins 1–3 are antimicrobial peptides with broad antimicrobial activity, including anti-HIV activity [45,54,79,80], and are important for the anti-HIV activity of neutrophils [41]. Our finding of reduced α-defensin 1–3 secretion by neutrophils after SCFA treatment may indicate reduced HIV inactivation potential. 

Furthermore, we detected a delay in NET release after HIV stimulation when neutrophils were challenged in the presence of SCFAs. In contrast to a previous report [81] showing increased NET release after neutrophil treatment with SCFAs in the mM concentration range, we did not observe any significant differences after SCFA treatment in the absence of HIV stimulation. The reason behind these disparate results remains to be determined but may be due to the different methodologies used to quantify NETs. We have recently demonstrated that neutrophils from the FRT and blood display direct anti-HIV activity through the release of NETs [40]. Therefore, our results suggest that tissue environments with pathological concentrations of SCFAs can reduce NET release and impair anti-HIV defense by neutrophils. Interestingly, while propionate delayed NET release in both younger and older women, butyrate and acetate preferentially affected NET release in neutrophils from younger women. The mechanisms responsible for these differences remain unknown but recognizing that the composition of the vaginal metabolome changes as women age, this finding may be relevant to understanding how anti-HIV protection changes with age. 

Another novel finding in our study is that we demonstrate that HIV stimulation of neutrophils in vitro induces the secretion of the chemokines IL-8, MCP-1, MIP1α, RANTES, MDC and MIG, and this effect is dampened in the presence of a high concentration of SCFAs, particularly butyrate. This finding adds to the evidence suggesting that SCFAs reduce the ability of neutrophils to respond to HIV stimulation. 

Lastly, our study has several limitations. While our results with blood neutrophils offer insight into how environments with high SCFA concentrations may modify neutrophil phenotype and function, future studies with neutrophils obtained from the genital tract of women with vaginal dysbiosis are needed to confirm our results. Further, we address the effects of individual SCFAs, but in vivo, we would expect a combination of SCFAs and different combinations depending on the microbiome alterations [32]. Studies that analyze the effects of vaginal secretions (with a known metabolome) on neutrophils are needed. We describe age-dependent effects; however, the sample size included in this study is limited, and the ages of the women recruited for our study were very polarized between younger women (18–35 years old) and older women (54–72 years old). Further studies with larger sample sizes are needed to confirm our results and to define changes in middle-aged and perimenopausal groups, also at high risk for infection in the US [82]. Finally, we have described a number of factors that are modified in a high-SCFA environment that could be relevant for inflammation and antiviral defense; however, additional factors and underlying mechanisms for the described changes remain to be investigated, such as, for example, the potential role of galectin-9 modifications in inflammatory cytokine/chemokine release and neutrophil recruitment [83,84,85,86].

## 5. Conclusions

Overall, our study demonstrates that pathological concentrations of SCFAs can induce phenotypic changes in neutrophils in an age-dependent manner, specifically butyrate and propionate, which differentially upregulate CD16, CD62L and CD54, classical markers of activation, maturation and aging. Furthermore, high concentrations of SCFAs reduce anti-HIV responses in neutrophils, including reduced secretion of the antimicrobial peptide α-defensin 1–3, reduced secretion of chemokines and delayed NET release following HIV stimulation. Our findings provide proof of concept that genital microbial alterations which induce an increase in SCFA concentrations may impair neutrophil physiological functions and reduce their antiviral potential.

## Data Availability

Not applicable.

## Supplementary Materials

The following supporting information can be downloaded at: https://www.mdpi.com/article/10.3390/cells11162515/s1, Figure S1: Gating strategy and survival of neutrophils after treatment with SCFAs; Figure S2: Effect of age on cytokine and chemokine release by neutrophils after treatment with pathological concentrations of SCFAs; Figure S3: Pathological concentrations of SCFA did not change basal NET release by unstimulated neutrophils.
