# Supplementary material for: Short-Chain Fatty Acids Impair Neutrophil Antiviral Function in an Age-Dependent Manner
Source: Cells. 2022 Aug 13;11(16):2515. doi: 10.3390/cells11162515 (PMC9406757; doi:10.3390/cells11162515)
Supplement: Supplementary file 1 [file cells-11-02515-s001.zip › cells-1756527-supplementary.pdf]

(a)

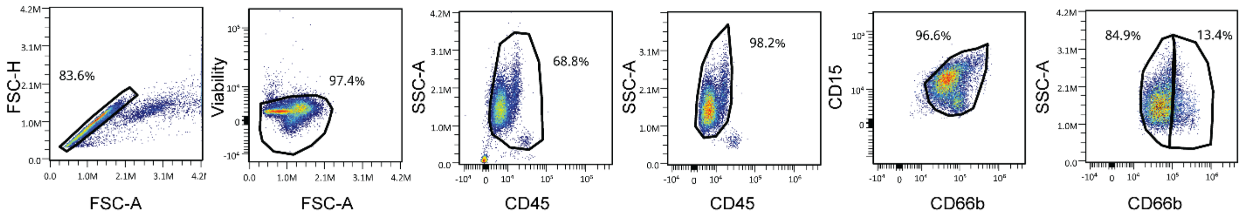

(b)

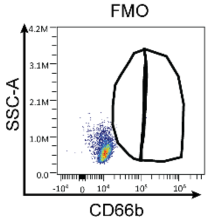

Figure S1. Gating strategy and viability of neutrophils after treatment with SCFAs. (**a**) Representative gating strategy to identify neutrophils after stimulation with SCFAs. Neutrophils were identified as CD45^{+}, SSC-A^{high}, CD15^{+} CD66b^{+} cells. (**b**) FMO for CD66b, gated on CD45^{+}.

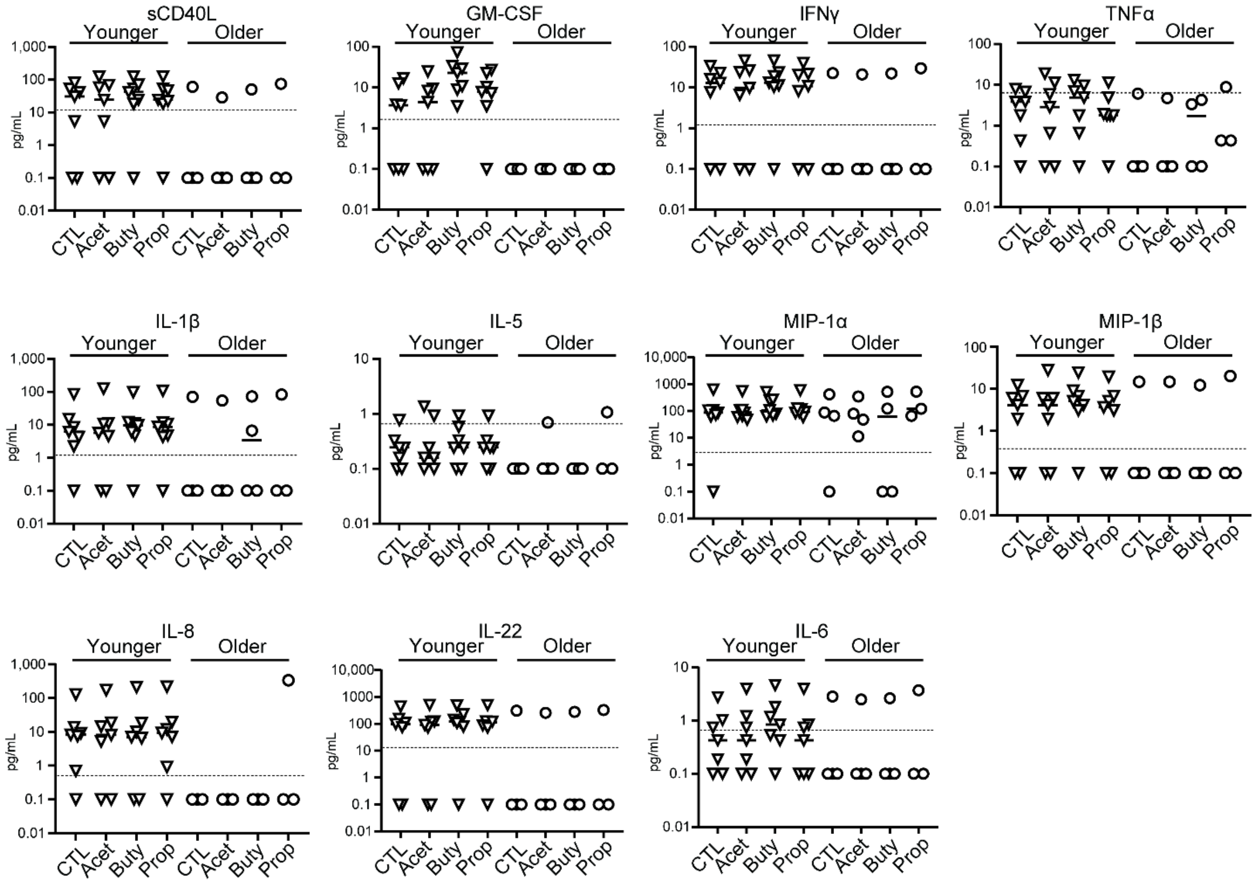

Figure S2. Cytokine and chemokine release by neutrophils after treatment with pathological concentrations of SCFAs. Neutrophils were incubated for 3 h in the presence or absence of SFCAs, and cell-free supernatants were used to quantify the concentration of cytokines and chemokines by Luminex. Each dot represents a different patient. Triangles: younger (*n* = 7); Circles: older (*n* = 4). Dotted line: limit of detection. CTL: control; Acet: acetate 25 mM; Buty: butyrate 25 mM; Prop: propionate 25 mM.

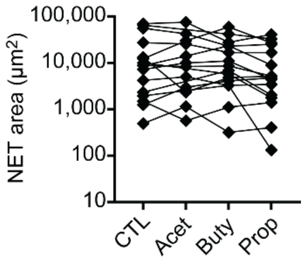

Figure S3. Pathological concentrations of SCFA did not change basal NET release by unstimulated neutrophils. Each dot represents a different patient (n = 17; younger = 8; older = 9). Kruskal–Wallis with Dunn's post-test was used to compare three or more groups. CTL: control; Acet: acetate 25 mM; Buty: butyrate 25 mM; Prop: propionate 25 mM.
